# OCT Evaluation of Retinal Parameters in Pediatric Gastritis Patients with Helicobacter Pylori

**DOI:** 10.14744/bej.2021.42104

**Published:** 2021-12-17

**Authors:** Mehmet Fatih Kocamaz, Gulseren Sahin, Ferda Ozbay Hosnut, Nesibe Gokce Kocamaz, Pinar Altiaylik Ozer, Ahmet Sengun

**Affiliations:** 1. Department of Ophthalmology, Bursa City Hospital, Bursa, Turkey; 2. Department of Pediatric Gastroenterology, Dr. Sami Ulus Maternity and Child Health and Diseases Training and Research Hospital, İstanbul, Turkey; 3. Department of Child Health and Diseases, Gemlik State Hospital, Bursa, Turkey; 4. Department of Ophthalmology, Ufuk University Faculty of Medicine, Ankara, Turkey

**Keywords:** Choroidal thickness, ganglion cell layer complex thickness, gastritis, optical coherence tomography, Helicobacter pylori, retinal nerve fiber layer thickness

## Abstract

**Objectives:**

The aim of this study was to investigate the effect of Helicobacter pylori (H. pylori) infection on choroidal thickness (CT), retinal nerve fiber layer (RNFL) thickness, and ganglion cell (GCL+IPL) complex thickness in childhood cases of gastritis.

**Methods:**

A total of 104 eyes of 52 children were included in the study. Two groups were created: 54 eyes of 27 H. pylori gastritis cases (Group 1) and 50 eyes of 25 gastritis without H. pylori cases (Group 2), as confirmed by an endoscopic biopsy. The mean subfoveal, submacular, and peripapillary CT, RNFL thickness, and GCL+IPL complex thickness was measured using spectral domain optical coherence tomography.

**Results:**

The mean subfoveal CT values were significantly higher in Group 1 compared with Group 2 (p=0.042). The mean submacular CT and peripapillary CT measurements of the eyes in Group 1 was greater than that of Group 2, but the difference was not statistically significant (p>0.05). There was also no statistically significant difference between the GCL+IPL complex or RNFL thickness values of the groups (p>0.05).

**Conclusion:**

H. pylori is a common gastrointestinal infectious agent with asymptomatic carriers in the population. The role of this agent in ocular pathologies in adult patients has been the subject of many recent studies, but secondary ocular findings in patients with H. pylori gastritis in childhood have not yet been investigated. The results of this study showed that the subfoveal CT value was significantly greater in children with H. pylori gastritis.

## Introduction

Helicobacter pylori (H. pylori) is a gram-negative, urease, catalase and oxidase-positive, curved, motile bacterium with 4-6 flagellas (1). H. pylori infected approximately 50% of the world population, although the incidence varies regionally and socioeconomically. H. pylori-induced gastritis can be diagnosed even at childhood. The incidence is between 3% and 10% in pediatric age group. Especially in developing countries, 2/3 of infected children encounter H. pylori until the age of two (2).

While children with chronic gastritis are usually asymptomatic, a group of patients may have dyspeptic complaints and recurrent abdominal pain (3). Infection often causes gastrointestinal complaints, but also causes extra-gastric complaints by creating chronic oxidative stress by a number of cytokines such as ınterleukin (IL)-1, IL-2, IL-6, IL-8 and tumour necrosis factor α (TNF-α) (4). Until now, many studies have demonstrated the extra-gastric associations of H. pylori such as cardiac pathologies, respiratory pathologies, central nervous system diseases, skin diseases, gastrointestinal system diseases, and some ocular diseases (5, 6).

The role of H. pylori in ophthalmological pathologies was first described by Mindel and Rosenberg in 1997 (7). The number of studies examining the relationship between ophthalmologic pathologies and H. pylori has increased due to the increasing interest over the years. The association of H. pylori infection with central serous chorioretinopathy (CSCR), blepharitis, glaucoma, and anterior uveitis have been shown in many studies (8-11). There are also some reports which suggest the bacterial eradication of H. pylori -especially in resistant cases- increases the treatment response of ophthalmic pathology (12).

The choroid has the most intense vascularity per square millimeter in the human body (13). CSCR is a clinical condition secondary to disruption of choriocapillaris, often involving both eyes asymmetrically (14). In many studies, it is stated that increase in the thickness of choroidal layers in high-definition-optical coherence tomography (HD-OCT) may be a sign of early deterioration of choroidal vessels in CSCR patients which leads to serous neurosensory detachment causing fluid accumulation among choroidal layers (14). At this point, vascular endothelial damage caused by some mediators released due to H. pylori colonization appears to be one of the accused factors in the etiology of CSCR within the pachychoroid disease spectrum (14).

The aim of this study was to investigate the retinal and uveal effects of H. pylori in pediatric cases with gastritis - which are currently increasing in the prevelance among childhood population.

## Methods

This study was designed as a multicenter, double-blind, prospective study. Ufuk University Faculty of Medicine Dr. Rıdvan Ege Research Hospital Non-Drug Clinical Research Ethics Committee approval was obtained (Date March 23, 2018-5). In addition, written informed consent was obtained from at least 1 parent of all children included in the study in accordance with the Helsinki Declaration of the World Medical Association (15).

One hundred and four eyes of 52 children with gastritis diagnosed between March and June 2018 were included in the study. The study groups consisted of patients aged 6–18 years who were referred to the Pediatric Gastroenterology Outpatient Clinic of Dr. Sami Ulus Maternity and Child Health and Diseases Training and Research Hospital with dyspeptic complaints. All participants underwent upper gastrointestinal endoscopy and gastric biopsy. Endoscopic gastric biopsy specimens obtained from the patients included in the study were scored according to the “Updated Sydney Classification System” evaluation criteria and were divided into two groups as gastritis with H. pylori and gastritis without H. pylori. Gastritis with H. pylori group also has H. pylori antigen positivity or urease positivity in feces. Group 1 was selected from patients who were accused of H. pylori in the etiology of gastritis, and the control group (Group 2) was selected from patients who were thought to have etiologic factors other than H. pylori despite the presence of gastric symptoms.

Patients under 18 years of age, who have no demonstrated ocular or systemic disease except gastritis; no history of ocular surgery and refractive error with spherical equivalent between –1.25 and +1.25 diopters were included in the study. The presence of any optical media opacity to affect HD-OCT images, and 5/10 or lower quality of HD-OCT imaging were excluded from the study. In addition, patients who had received antisecretory agents, antiinflammatory drugs and antibiotics in the past 3 months or who had H. pylori eradication in the last 1 year were excluded from the study.

All subjects underwent thorough ophthalmologic examination including visual acuity assesment with Snellen chart, intraocular pressure measurement with Goldmann applanation tonometer, biomicroscopy and dilated fundus examination by the same ophthalmologist (MFK), who is blind to the presence of H. pylori infection in the subject. In addition, choroidal thickness, retinal nerve fiber layer thickness, and ganglion cell layer (GCL) + inner plexiform layer (IPL) complex thickness (Carl Zeiss Meditec, Model 400, Dublin, USA, Version 8.1.0.117) were calculated by spectral domain OCT (Cirrus HD-OCT). All choroidal thickness measurements with OCT were performed between 9 AM and 11 AM to avoid diurnal variation (16).

Optical disc cube 200 × 200, Macular thickness: Macular cube 512 × 128, Ganglion cell: Macular cube 512 × 128, High definition images (EDI): HD 5 Line raster modes were used in vertical and horizontal axes for optic disc and macular measurements. Measurements taken in HD 5 line Raster mode were used to calculate choroidal thickness values. Thickness of the choroid was accepted as the distance between the outer edge of the hyperreflective image of the retinal pigment epithelium (RPE) and the hyperreflective inner part of the sclera. Measurements of the choroidal thickness were performed manually.

**Figure 1.**
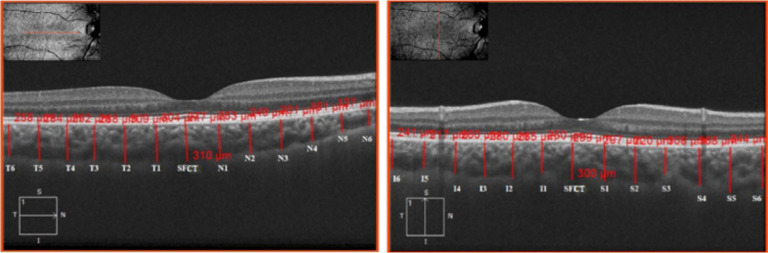
Example of subfoveal choroidal thickness and average submacular choroidal thickness measurement of a patient in Group 2 with HD-OCT, horizontal section and vertical section, respectively. SFCT: subfoveal choroidal thickness. T1, T2, T3, T4, T5, T6 show measurements of temporal region; N1, N2, N3, N4, N5, N6 show measurements of nasal region; I1, I2, I3, I4, I5, I6 show measurements of inferior region; S1, S2, S3, S4, S5, S6 show measurements of superior region at 500 micron intervals from the central fovea.

Subfoveal choroidal thickness values of the volunteers were calculated manually for each eye from the center of the fovea (Fig. 1). Subsequently, for the submacular choroidal thickness (SMCT), measurements were performed from the inferior, nasal, temporal, and superior quadrants from center of the fovea to 3000 μm at 500 μm diameter. Since different methods have been described in the literature to obtain more accurate results, not only horizontal but also vertical quadrants were taken into account when calculating mean SMCT. In this study, we developed and used a 25 points-measurement method, instead of the model, which was described by Tenlik et al. (17), made from 500 to 3000 μm (5 points) in the horizontal and vertical cross-section (Fig. 1). Mean SMCT was calculated by taking the average of all horizontal, vertical and subfoveal measurements.

**Figure 2.**
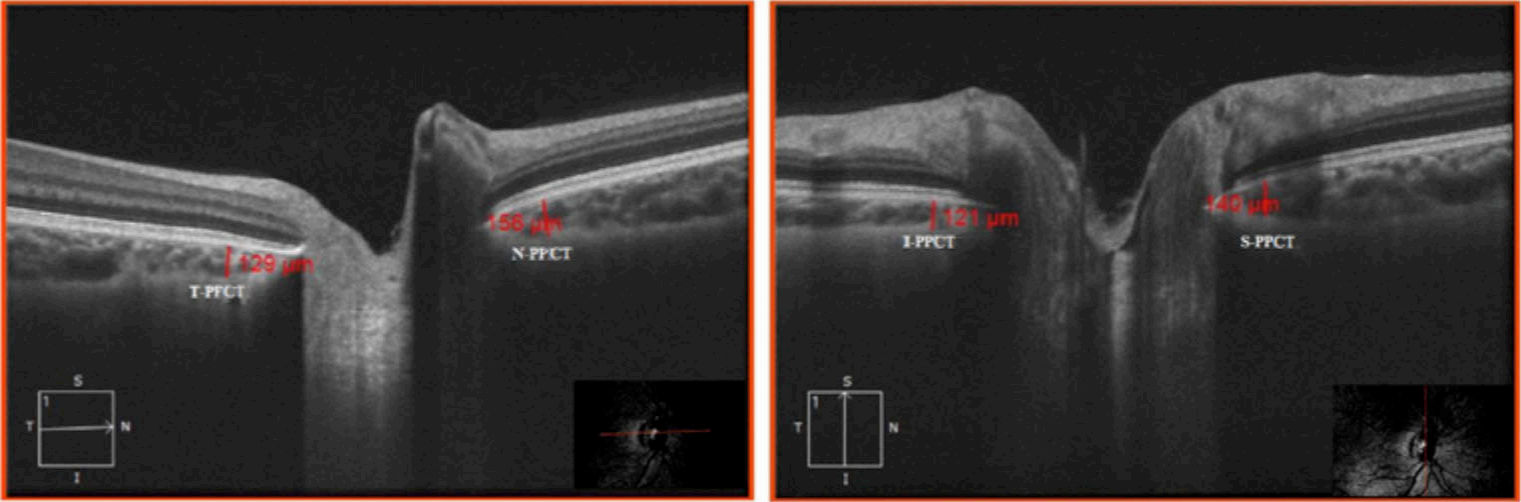
Example of peripapillary choroidal thickness measurement of a patient in Group 2 with high definition OCT images, horizontal section and vertical section, respectively. PPCT: peripapillary choroidal thickness. T-PPCT shows the measurement of temporal region; N-PPCT shows the measurement of nasal region; I-PPCT shows the measurement of inferior region; S-PPCT shows the measurement of superior region at 500 micron distance from the end point of the RPE at optic disc.

For the peripapillary choroidal thickness (PPCT), measurements performed manually at 500±8 μm distance in each of the 4 quadrants (superior, inferior, nasal, temporal) based on RPE which was ended at the optic nerve level horizontally and vertically. Mean values from 500 μm measuring points from the end of RPE were used (Fig. 2).

The ganglion cell analysis mode in the Cirrus HD-OCT device (Carl Zeiss Meditec, Model 400, Dublin, USA, Version 8.1.0.117) obtains the sum of the GCL + IPL thickness using data from the 512 × 128 macular cube analysis. In the current study, mean GCL + IPL complex thickness, minimum value of GCL + IPL complex thickness, and thickness values of 6 segments were recorded as superonasal, superior, superotemporal, inferotemporal, inferior and inferonasal regions separately for each eye. Thickness values were taken as micron (μm) and numerical data were coded and transferred to computer program.

For retinal nerve fibre layer (RNFL) thicknesses, measurements were performed in “Optic Disc Cube 200 × 200” mode. RNFL screening results were evaluated. Average, superior, temporal, nasal, and inferior RNFL thickness values were calculated from 5 regions. In the statistical analysis, all RNFL thickness values were taken separately for each eye.

All patients’ choroidal thickness results were evaluated as double-blind by two independent observers (MFK, PAO). The correlation between the observers was statistically analyzed. After the ophthalmologic examination and OCT measurements were completed, cases were divided into two groups according to the information obtained from pediatric gastroenterology department according to the etiology of gastritis. Fifty four eyes of 27 patients with H. pylori gastritis confirmed by biopsy were grouped as Group 1 and 50 eyes of 25 patients with non-H. pylori factors in the etiology of gastritis were grouped as Group 2.

Data were analyzed using IBM SPSS Statistics 17.0 (IBM Corporation, Armonk, NY, USA). While comparison of Group 1 and Group 2 was performed, and results were considered statistically significant when p<0.05. However, Bonferroni Correction was performed in the present study to check for Type I error in all possible multiple comparisons. While the statistical analysis was performed, Shapiro–Wilk test and homogeneity of variance were investigated using Levene test. Descriptive statistics; for continuous numerical variables, mean ± standard deviation or median (25–75) percentages were shown, and categorical variables were expressed as number of cases and (%). The subfoveal, mean submacular and mean PPCT measurements between the observers were evaluated using the Class Correlation Coefficient and 95% confidence intervals. The significance of the difference in terms of continuous numerical variables where parametric test statistic assumptions were not provided was examined with Mann–Whitney U test, while The significance of the difference between the groups in terms of mean values was evaluated by Student’s t-test. The categorical data were evaluated using Fisher’s Exact Probability Test and the Continuity Corrected Chi-square test if the expected frequency was <5 in 2×2 cross tables.

The significance of the difference in terms of clinical measurements where parametric test statistic assumptions were not provided was evaluated by Wilcoxon sign test.

## Results

Mean age and female/male distribution among Group 1 and Group 2 were found to be similar (p=0.428, p=0.999, respectively) (Table 1).

**Table 1.** Demographics of cases in our study

	***H. pylori* positive (Group 1) (n=27)**	***H. pylori* negative (Group 2) (n=25)**	**p**
Age (years)	13 (10–15)	12 (10–14.5)	0.428^‡^
Male/Female Ratio (n/n)	8/19	7/18	0.999^†^
Male, n (%)	8 (29.6)	7 (28.0)	>0.01
Female, n (%)	19 (70.4)	18 (72.0)	>0.01

^‡^Mann-Whitney U test; ^†^Chi-square test with contunuity correction,

There was no significant differences among groups in terms of mean best corrected visual acuity, mean intraocular pressure values, spherical equivalents of mean refraction values. RNFL thickness, central subfield thickness, macular cube volumes and GCL + IPL complex thickness were compared among Group 1 and 2. There were no significant differences with Mann–Whitney U test according to Bonferroni Correction in terms of sectoral and average RNFL thickness values between Group 1 and Group 2 (p>0.05) (Table 2). There were also no significant differences between the groups in terms of central subfield thickness and macular cube volumes compared by Student’s t-test according to Bonferroni Correction (p=0.243 and p=0.401, respectively). Among groups, there was no significant difference in terms of mean and sectoral GCL + IPL complex thickness values compared by Student’s t-test and Bonferroni Correction (p>0.05) (Table 2).

**Table 2.** Comparison of RNFL thickness, GCL + IPL complex thickness, Central subfield thickness and Macular cube volume measurements in Study Groups

OCT parameters (mean of right and left eyes in each Group)*	***H. pylori* positive (Group 1)**	***H. pylori* negative (Group 2)**	**p**
Average RNFL thickness (μm)	99.0 (92.0–110.5)	93.5 (90.5–100.0)	0.068^‡^
Superior RNFL thickness (μm)	131.5 (120.5–138.5)	127.0 (113.0–133.0)	0.062^‡^
Nasal RNFL thickness (μm)	72.0 (67.0–87.0)	68.0 (65.2–77.5)	0.107^‡^
Inferior RNFL thickness (μm)	126.5 (109.5–148.0)	122.0 (112.7–134.0)	0.621^‡^
Medial RNFL thickness (μm)	67.0 (61.5–74.0)	66.5 (58.5–70.7)	0.452^‡^
Central subfield thickness (μm)	247.1±15.1	240.6±23.4	0.243^#^
Macular cube volume (mm^3^)	282.5±12.0	279.8±10.3	0.401^#^
Mean GCL+IPL complex thickness (μm)	84.9±6.0	84.2±5.6	0.671^#^
Minimum GCL+IPL complex thickness (μm)	82.5 (80.0–85.0)	82.0 (77.5–85.0)	0.458^‡^
Superior GCL+IPL complex thickness (μm)	85.6±6.1	85.3±5.5	0.866^#^
Superonasal GCL+IPL complex thickness (μm)	86.2±6.2	85.4±6.9	0.661^#^
Inferonasal GCL+IPL complex thickness (μm)	85.6±6.5	85.0±5.8	0.765^#^
Inferior GCL+IPL complex thickness (μm)	84.7±6.7	83.5±5.7	0.491^#^
Inferotemporal GCL+IPL complex thickness (μm)	84.8±6.4	83.6±6.2	0.484^#^
Superotemporal GCL+IPL complex thickness (μm)	82.4±5.7	82.8±5.5	0.783^#^

^‡^Mann-Whitney U test according to Bonferroni Correction, p<0.05 was statistically significant. ^#^Student’s t test according to Bonferroni Correction, p<0.05 was statistically significant. RFNL: Retinal nerve fiber layer; GCL: Ganglion cell layer; IPL: Inner plexiform layer; OCT: Optical coherence tomography.

Choroidal thickness values are compared among Group 1 and 2, mean subfoveal choroidal thickness of individuals in Group 1 were calculated significantly higher than those in Group 2. (p=0.042) Mean SMCT and mean PPCT values of these parameters for both eyes are compared, no significant difference was seen among Group 1 and Group 2 (p>0.05) (Table 3).

**Table 3.** Mean subfoveal, mean submacular and mean peripapillary choroidal thickness values of H. pylori positive gastritis group and H. pylori negative gastritis group

	**Mean Subfoveal choroidal thickness of both eyes**	**Mean peripapillary choroidal thickness of both eyes**	**Mean submacular choroidal thickness of both eyes**
*H. pylori* positive (Group 1)	376.0 (315.5–431.0)	166.5 (138.5–185.0)	318.0 (270.0–344.0)
*H. pylori* negative (Group 2)	328.0 (297.75–367.0)	148.5 (122.5–186.25)	289.0 (263.75–310.75)
p		0.042^‡^	0.327^‡^	0.206^‡^

^‡^Mann-Whitney U test according to Bonferroni Correction, p<0.05 was statistically significant.

There was a high compatibility between the first and second observers in terms of subfoveal choroidal thickness, mean SMCT and PPCT measurements (p<0.001).

## Discussion

H. pylori is the most common gastrointestinal infection agent in the world (18). These bacteria, which causes both gastric and extra-gastric pathologies, are a highly asymptomatic agent in the community (19).

Vascular involvement is the most common extra-gastric involvement of H. pylori described in the literature (20). The supposed pathophysiologic mechanism underlying extra-gastric pathologies of H. pylori is the possible inflammatory and immune response induced by it’s gastric colonization (6). The severity of the condition depends not only on colonization, but also on virulence of the bacteria and resistant strains. Because H. pylori lipopolysaccharides affect the immune system with a number of virulence factors such as urease and heat shock protein release (21). These substances activate T cells by affecting the macrophages of lamina propia in the stomach. The result is inflammatory cytokines such as IL-1, IL-6, IL-8, and TNF-α (21). Proinflammatory cytokines such as ILs released from infected gastric epithelial cells and histamine released by mast cell degranulation also mediate inflammation (22). Tissue damage may also occur due to the antigenic similarity relationship between H. pylori and non-digestive tissues (23). Free oxygen radicals and lipid peroxide derivatives play a key role in inflammatory tissue damage. Furthermore, H. pylori mediates vasodilatation, inflammation and immune modulation by increasing serum nitric oxide levels (24).

Systemic H. pylori colonization is not only limited to gastrointestinal tissues. It was shown that H. pylori can colonize in the middle ear and eye (25). The role of H. pylori in ophthalmologic pathologies in adult patients has been the subject of many studies. There are reports of association with glaucoma, idiopathic CSCR, ocular adnexal mucosa-associated lymphoid tissue lymphoma, Sjogren’s sydrome, blepharitis and uveitis (6, 9).

Studies on this microorganism, which has recently gained a popularity among ophthalmologists, mainly focus on CSCR. H. pylori is regarded as a possible risk factor in this disease, which is a part of pachychoroid disease spectrum with increased choroidal thickness. There are some authors who emphasize that H. pylori eradication is necessary in the treatment of CSCR cases (26, 27). The “Pachychoroid Disease Spectrum” group, which is thought to develop secondary to choroidal inflammation and where monitoring with OCT is of great importance, is the common name of a series of chorioretinopathies (28). The common clinical feature of this group of diseases including CSCR, pachychoroid pigment epitheliopathy and idiopathic polypoidal choroidal vasculopathy is the increase in choroidal thickness. Among these patients, H. pylori prevalence was reported to be 39.7–68.75% in patients with CSCR and diffuse retinal epitheliopathy. In the light of these data, H. pylori is now listed among the risk factors for CSCR, which is included in the pachychoroid disease group with increased choroidal thickness (29). Interactions between endothelium and bacteria in patients with H. pylori positivity cause widespread vascular damage, occlusion and disruption of microcirculation. This condition also occurs in choroidal microcirculation and may cause ischemia, resulting in CSCR (12).

In ophthalmology practice, measurements are made from reverse images obtained by the use of OCT, while images obtained with EDI mode available in many OCT devices allow the measurement of choroidal thickness at the micron level, even manually. With the help of OCT, normal and pathological values of choroidal and retinal layers that may vary among populations and in many systemic diseases have also been the subject of many studies. The increase in choroidal thickness detected in diseases such as diabetes mellitus, hypertension, obstructive sleep apnea, systemic lupus erythematosus, and rheumatoid arthritis are some examples indicating the change in choroidal blood flow in systemic conditions (30-34).

Although there are many studies among adults in the literature, there are limited studies investigating choroidal thickness in the pediatric age group. In this age group, secondary ocular findings in patients with H. pylori gastritis have not been previously studied, either.

In a study conducted by Park and Oh in 48 eyes of 48 volunteers, the mean age was 6.7±1.9, while the subfoveal choroidal thickness was found as 346 micrometers (35). It was stated that this value was negatively correlated with the age of the volunteers (35). In another study by Margolis and Spaide, performed among adults, measurements were made at 500 μm intervals on the 6 mm horizontal line passing through the fovea. It was found that the choroidal thickness was the highest in the subfoveal area and decreased gradually as it moved away from the fovea, it was thinner in the nasal and the thinnest at the end of the image obtained. This is due to the fact that, despite the high metabolic activity of the fovea, there is no direct blood flow from the retinal circulation and the thickness of the subfoveal choroid is higher (36).

H. pylori is a risk factor for CSCR, associated with an increase in choroidal thickness and included in the pachychoroid disease group. In a study previously performed by Atılgan et al., it was found that there was no significant increase in choroidal thickness values in the patients with H. pylori positivity in adults compared to the H. pylori negative patients (37). In another study by Horozoglu et al., it was observed that there was no increase in choroidal thickness values in H. pylori positive patients without CSCR (38). Can et al. reported that choroidal thickness values were found to be increased in adult patients with H. pylori infection and regression of choroidal thickness with treatment was shown (39). Contrary to the controversial results in the studies conducted in the adult age group, we think that the main factor in the formation of the statistical difference arises from the fact that aging and decreasing vascular elasticity and compliance. Vascular elasticity and compliance decreases with age at different levels (40). Thus, different choroidal thickness values may be obtained among different aged study groups of various studies.

In our study, mean subfoveal computed tomography values of the patients with H. pylori positivity were significantly higher than patients without H. pylori infection. It is known that there is a positive correlation between the severity of inflammation and choroidal thickness. Many previous studies have reported that choroidal thickness increases significantly in cases that increase choroidal inflammation (41). Depending on this previous knowledge, the difference between subfoveal choroidal thickness values in our study was thought to be due to increased inflammatory factors by H. pylori positivity.

## Conclusion

Our study was performed in a prospective, double blind manner to prevent any researcher bias. The manual choroidal thickness measurements were repeated by two different observers for the elimination of personal factors and the interobserver consistency was repeated. There was a high interobserver compatibility (p<0.001). Major limitation of our study is the limited number of patients. More extensive studies with larger groups and with other technologies are necessary to establish the effects of H. pylori and other chronic infections on ocular tissues in all age groups.

H. pylori infection in childhood is thought to be a risk factor for increased choroidal thickness due to it’s vasculopathy potential similar to that in adult cases. In the light of these data, although carried on a small number of patients, we think that our study has a great contribution to the literature because it was performed in pediatric patients, and it was the first study that showed, H. pylori could cause choroidal thickness increase in this age group as well.

The clinical significance of the increase in choroidal thickness in the pediatric age group is unknown. However, whether this situation is a risk factor for pachycoroid spectrum diseases in older ages or not will be clarified with more extensive studies in the future. In the light of these scientific data, we think that in the presence of a history of pediatric gastritis in which H. pylori was documented as an etiological agent, it would be appropriate to follow-up patients in terms of choroidal pathologies.

### Disclosures

**Ethics Committee Approval:** The present study was approved by the local ethics committee and informed consent was obtained from all participants or their parents or legal representatives. This study was conducted in accordance with the Declaration of Helsinki.

**Peer-review:** Externally peer-reviewed.

**Conflict of Interest:** None declared.

**Authorship Contributions:** Involved in design and conduct of the study (MFK, PAO, AS); preparation and review of the study (MFK, PAO, GS); data collection (MFK, NGK, GS, FOH); and statistical analysis (MFK, PAO).
